# Supplementary material for: Impaired microglia-mediated synaptic pruning in the nucleus accumbens during adolescence results in persistent dysregulation of familiar, but not novel social interactions in sex-specific ways
Source: bioRxiv. 2023 May 3:2023.05.02.539115. Preprint. [Version 1] doi: 10.1101/2023.05.02.539115 (PMC10187149; doi:10.1101/2023.05.02.539115)
Supplement: Supplement 1 [file NIHPP2023.05.02.539115v1-supplement-1.pdf]

bioRxiv preprint doi: https://doi.org/10.1101/2023.05.02.539115; this version posted May 3, 2023. The copyright holder for this preprint (which was not certified by peer review) is the author/funder, who has granted bioRxiv a license to display the preprint in perpetuity. It is made available under aCC-BY 4.0 International license.

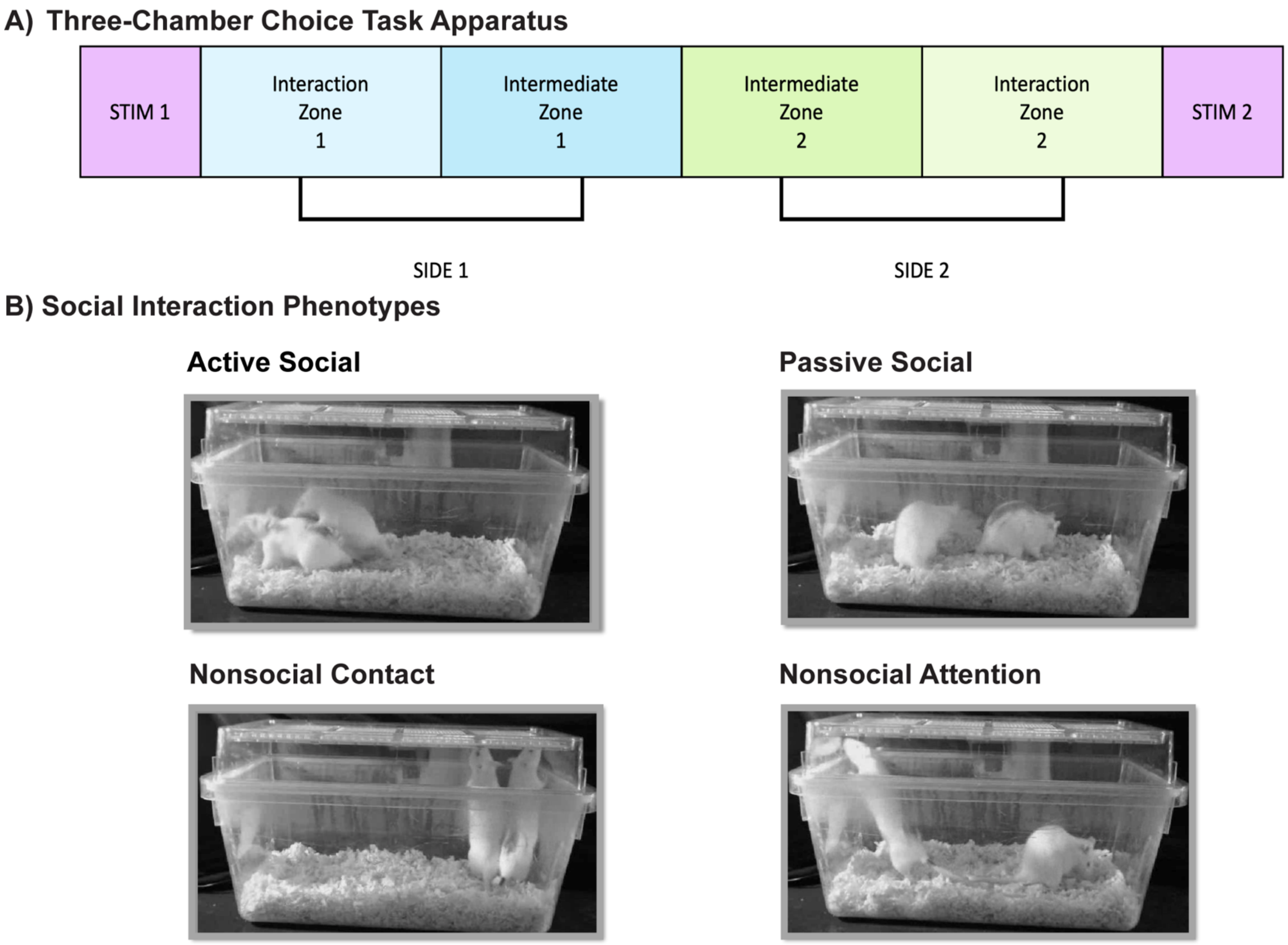

Supp. Fig. 1. *(A) Schematic of choice tasks apparatus. %exploration= time in interaction zone/time in target side. (B) Representative images of interaction metrics*
